# A RISC-V Processor with Area-Efficient Memristor-Based In-Memory Computing for Hash Algorithm in Blockchain Applications

**DOI:** 10.3390/mi10080541

**Published:** 2019-08-16

**Authors:** Xiaoyong Xue, Chenzedai Wang, Wenjun Liu, Hangbing Lv, Mingyu Wang, Xiaoyang Zeng

**Affiliations:** 1 State Key Laboratory of ASIC and System, Fudan University, Shanghai 201203, China; 2 Key Laboratory of Microelectronics Devices and Integrated Technology, Institute of Microelectronics of the Chinese Academy of Sciences, Beijing 100029, China

**Keywords:** in-memory computing, memristor, RISC-V, Internet of things, blockchain

## Abstract

Blockchain technology is increasingly being used in Internet of things (IoT) devices for information security and data integrity. However, it is challenging to implement complex hash algorithms with limited resources in IoT devices owing to large energy consumption and a long processing time. This paper proposes a RISC-V processor with memristor-based in-memory computing (IMC) for blockchain technology in IoT applications. The IMC-adapted instructions were designed for the Keccak hash algorithm by virtue of the extendibility of the RISC-V instruction set architecture (ISA). Then, a RISC-V processor with area-efficient memristor-based IMC was developed based on an open-source core for IoT applications, Hummingbird E200. The general compiling policy with the data allocation method is also disclosed for the IMC implementation of the Keccak hash algorithm. An evaluation shows that >70% improvements in both performance and energy saving were achieved with limited area overhead after introducing IMC in the RISC-V processor.

## 1. Introduction

Internet of things (IoT) refers to the network of different physical devices, which enables them to collect and exchange data [1,2]. With the development of telecommunication, computers, and integrated circuits, IoT is being increasingly applied in commercial fields such as modern agriculture, driverless vehicles, smart cities, etc., which promise to become vital parts of global economics [3]. However, as billions of IoT devices are connected to the continuously growing networks, security appears to be a major concern. IoT devices collect a great amount of private information, which is vulnerable to attacks if not well protected. Moreover, most of the devices are resource-constrained and, thus, heavy cryptographic approaches are difficult to implement.

**Figure 1:**
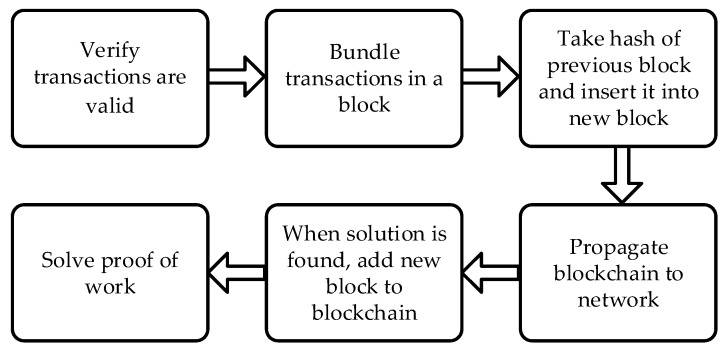
Bitcoin mining process using blockchain technology.

Recently, a trend emerged to exploit the blockchain technology in IoT devices for information security and data integrity [4]. The blockchain is a peer-to-peer (P2P) ledger which was first used in the Bitcoin cryptocurrency for economic transactions [5]. Bitcoin users that are known by a changeable public key generate and broadcast transactions to the network to transfer money. These transactions are pushed into a block by users. Once a block is full, the block is appended to the blockchain by performing a mining process. To mine a block, some specific nodes known as miners try to solve a cryptographic puzzle named proof of work (POW), and the node that solves the puzzle first mines the new block to the blockchain, as shown in Figure 1. Because of its distributed, secure, and private nature, the blockchain can enable secure messaging between devices in an IoT network. In this approach, the blockchain treats message exchanges between devices similar to financial transactions in a Bitcoin network. To enable message exchanges, devices leverage smart contracts which model the agreement between two parties. The distributed datasets maintained by blockchain technology also allow the data to be safely stored by different peers, and people are not required to entrust IoT data produced by their devices to centralized companies [6]. Moreover, the blockchain technology lowers the cost of the deployment of the IoT devices and makes it safe and easy for users to pay for the data on IoT devices [7].

However, the hardware for mining in IoT devices has to be lightweight, low-cost, and energy-efficient to adapt the blockchain technology. IoT devices are often deployed in nonhuman conditions to a great extent and are powered through batteries, calling for extremely low cost and low energy consumption [8]. However, when using a general processor, i.e., central processing unit (CPU) or graphics processing unit (GPU), to implement the blockchain, it is likely to consume too much energy, resulting in frequent recharging or a short battery lifetime. Resorting to a conventional application-specific integrated circuit (ASIC) or coprocessor can help to reduce energy consumption and improve speed, but will induce considerable area cost [9].

In-memory computing (IMC) provides a promising alternative. In a general processor, the data transfer on the bus between the central processing unit (CPU) and the memory leads to large power consumption and limited performance, i.e., memory bottleneck. To address this issue, IMC modifies the memory to be able to perform some regular logic operations such as AND, OR, and exclusive or (XOR) [9]. Especially for data vectors with large bit width, IMC can accomplish the AND/OR/XOR operation in one read access, saving both execution time and power consumption. Static random-access memory (SRAM) can be employed in IMC, but its cell size is too large with 6–10 transistors and it also needs constant power to hold the data, incurring considerable area cost and standby power [10].

Emerging memory technologies, especially memristors, feature a simple cell structure, high density, three-dimensional (3D) stackability, good compatibility with complementary metal–oxide–semiconductor (CMOS) processes, and non-volatility [11]. Recently, memristors were investigated to realize IMC using a one-transistor-one-memristor (1T1R) array accompanied by modified peripheral circuits [12]. However, it is still difficult to rely on memristor-based IMC alone to implement the hash algorithm in blockchain technology. A processor is still required to perform the data allocation, as well as other complexed logic operations. For resource-limited IoT devices, the processor should be flexible to support memory computation instructions while incurring small power consumption and area cost. Thanks to its simplicity, scalability, fast speed, and low power, the RISC-V processor is believed to be competent for the abovementioned requirements [13,14]. The instruction set architecture (ISA) of the RISC-V is designed to avoid over-architecting, while supporting command extension to achieve high flexibility [13]. Nevertheless, for the practical integration of IMC in RISC-V, the corresponding compiling policy and data allocation method still need specific consideration.

This paper proposes a RISC-V processor with memristor-based IMC for blockchain technology in IoT applications. The IMC-adapted instructions are designed for the Keccak hash algorithm by virtue of the extendibility of the RISC-V ISA. Then, a RISC-V processor with area-efficient memristor-based IMC is developed based on the open-source core, Hummingbird E200. The general compiling policy with data allocation method is also disclosed for the Keccak hash algorithm. An evaluation shows that remarkable improvements in performance and energy consumption are achieved with limited area overhead after introducing IMC.

The rest of the paper is organized as follows: Section 2 gives the IMC-adapted ISA design for the hash algorithm. Section 3 describes the RISC-V processor architecture with IMC and the implementation of IMC. Section 4 provides the policy for compiling and data allocation. Section 5 presents the evaluation, and Section 6 concludes this paper.

## 2. IMC-Adapted ISA Design for Hash Algorithm

### 2.1. Hash Algorithm in Blockchain Technology

A blockchain is literally a chain of blocks, each of which has a block header containing the hash value of its parent block to ensure the integrity of the chain [5]. With the rapid development of both computer hardware and software, traditional hash algorithms like Message-Digest algorithm 4 (MD4), Message-Digest algorithm 5 (MD5), and Secure Hash Algorithm 1 (SHA-1) were cracked. Therefore, the United States (US) National Institute of Standards and Technology (NIST) selected the Keccak sponge function family as the third-generation secure hash algorithm (SHA-3) to ensure the security of hash algorithms [15,16].

Keccak or SHA-3 shares a structure involving sponge functions with different parameters. The default Keccak sponge function works on a 1600-bit state array, which is logically a three-dimensional array with a row and column width of five and a lane width of 64. The array is often denoted as [x][y][z] in GF(2), where 0 ≤ *x* ≤ 4, 0 ≤ *y* ≤ 4, and 0 ≤ *z* ≤ 63.

The process of the Keccak sponge function consists of two phases, i.e., the absorbing phase and the squeezing phase. In the absorbing phase, the *r*-bit input blocks are XORed into the first *r* bits of the state, interleaved with a permutation called Keccak-*f* permutation; when all input blocks are processed, the sponge construction switches to the squeezing phase. In the squeezing phase, the first *r* bits of the state are returned as output blocks, interleaved with Keccak-*f* permutation; the number of output blocks is chosen at will by the user. Here, the value *r* is the bit rate. The process of the Keccak sponge function is actually an iteratively executed Keccak-*f* permutation, which takes most of the executing time. By default, 24 Keccak-*f* permutations take place for one permutation of sponge function.

Keccak-*f* permutation consists of five steps, which are the θ step, ρ step, π step, χ step, and ι step. The corresponding calculations of the five steps are shown in Equations (1)–(7). More detailed information for the algorithm can be found in Reference [17]. Table 1 summarizes the main processes performed in the five steps where the calculations of large vectors are hopefully implemented by the IMC.

(1) $$a\left[x\right]\left[y\right]\left[z\right]\leftarrow a\left[x\right]\left[y\right]\left[z\right]+\overset{4}{\underset{y^{\prime }=0}{\sum }}a\left[x-1\right]\left[y^{\prime }\right]\left[z\right]+\overset{4}{\underset{y^{\prime }=0}{\sum }}a\left[x+1\right]\left[y^{\prime }\right]\left[z-1\right].$$

(2) $$a\left[x\right]\left[y\right]\left[z\right]\leftarrow a\left[x\right]\left[y\right]\left[z-\frac{1}{2}\left(t+1\right)\left(t+2\right)\right].$$

(3) $${\left(\begin{array}{cc} 0 & 1\\ 2 & 3 \end{array}\right)}^{t}\left(\begin{array}{c} 1\\ 0 \end{array}\right)=\left(\begin{array}{c} x\\ y \end{array}\right),\;0\leq t\leq 24\;\mathrm{or}\;x=y=0,\;t=-1.$$

(4) $$a\left[x\right]\left[y\right]\leftarrow a\left[x^{\prime }\right]\left[y^{\prime }\right],\;\left(\begin{array}{c} x\\ y \end{array}\right)=\left(\begin{array}{cc} 0 & 1\\ 2 & 3 \end{array}\right)\left(\begin{array}{c} x^{\prime }\\ y^{\prime } \end{array}\right).$$

(5) a[x]←a[x]+(a[x+1]+1)a[x+2].

(6) $$a\left[0\right]\left[0\right]\leftarrow a\left[0\right]\left[0\right]+R_{i}.$$

### 2.2. IMC-Adapted ISA Design 

Before proposing the RISC-V processor with IMC for SHA-3, the characteristics hidden in Keccak calculations and how to adapt the ISA to support the IMC are investigated. Many operations in SHA-3, especially the sheet and plane logic operations, require frequent memory access and can be greatly optimized by adopting IMC, since they are 320 bits long while a processor is often 32-bit or 64-bit. RISC-V ISA is highly extendable and provides the users with four custom operations in its base instruction set and long custom instruction sets to be defined in the future [12]. To improve SHA-3 performance, only a few IMC instructions are needed; thus, this work employs the four custom operations to adapt IMC. The long custom instruction sets are reserved for more IMC operations as needed.

The operations in Keccak-*f* permutation can be classified into four different types, which are (1) long bitwise logic operations (both 64 bits and 320 bits), (2) 64-bit shift operations on a 320-bit binary string, (3) 64-bit data copying, and (4) operations on one 64-bit binary string. For these four types of operations, the first three can be easily implemented by IMC technology. Based on the above analysis, three kinds of IMC operations are adopted, including 320-bit bitwise logic operations (XOR, OR, and AND), 64-bit shift operation (SHIFT), and 64-bit data copying operation (CP). In addition, an operation that copies 64-bit data to all columns in another row address (copy to all columns, CPA) is needed for data allocation purposes (see Section 4). CPA operations are also needed in the θ step and χ step for data allocation purposes. Table 2 shows the IMC operations involved in different steps of Keccak-*f* permutation.

Table 3 shows the detailed IMC instruction definition. The IMC logic instructions including XOR, OR, and AND perform the 320-bit logic operation with operands from addresses (BA + A1) and (BA + A2), and store the results in (BA + A0). A0, A1, and A2 are addresses either from immediate or registers, depending on 3-bit I/R, and BA is an address from a register. SHIFT instruction performs the 64-bit circular right shift on (BA + A1) by SA[6:0] amount and stores the result in (BA + A0). A0 and A1 are addresses either from immediate or from registers, depending on 2-bit I/R. The addresses used in 320-bit operations are all 9-bit row addresses; thus, only 9 bits in the address are valid. The normal read loads the 32-bit data from memory address (rs + Imm[11:0]) to register rd. The normal write stores the 32-bit word data in register rs2 to memory address (rs1 + Imm[11:0]). For CP and CPA instructions, when Flag = 0, the data in memory address (BA + A1 + Col[2:0]) are copied to address (BA + A2 + Col[5:3]) for CP; when Flag = 1, the data in memory row address (BA + A1 + Col[3:0]) are copied to all the columns in row address (BA + A2) for CPA. The reserved bits in the IMC-adapted ISA can be used for more functions if necessary.

## 3. RISV Processor with IMC

### 3.1. Processor Architecture

RISC-V foundations introduced a few open-source RISC-V processor cores. This work chose Hummingbird E200 as the original processor because it was designed for IoT applications and optimized for low power and area costs [18].

**Figure 2:**
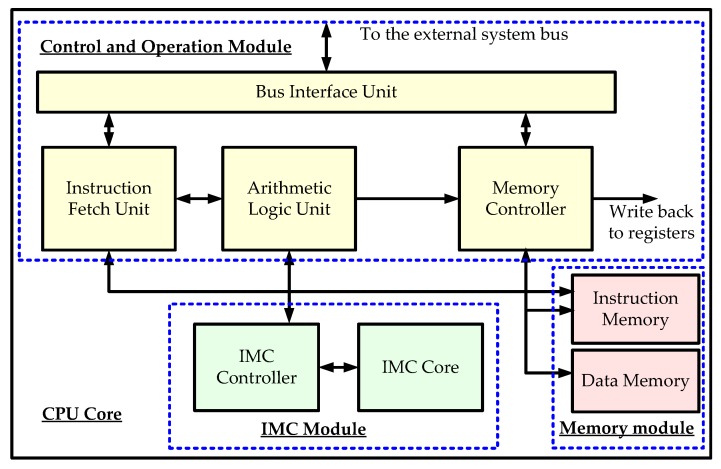
Modified RISC-V processor core with in-memory computing (IMC).

The original Hummingbird E200 processor employs two static random-access memories (SRAMs) as working memories, one for instructions and the other for data. This work adds an additional memory module, i.e., the IMC module, which includes an IMC core based on a memristor and a customized IMC controller to interact with the control and operation module (COM) in the CPU core, as shown in Figure 2. Some modifications are also made inside the processor without changing the original functions; thus, the generality is not destroyed after adding IMC functions. The memory controller is not reused for the IMC module because it has more functions than a traditional SRAM. Therefore, a separate controller is designed inside the IMC module (as discussed in Section 3.2).

### 3.2. IMC Implementation

#### 3.2.1. IMC Core Architecture and Assistant Logic

**Figure 3:**
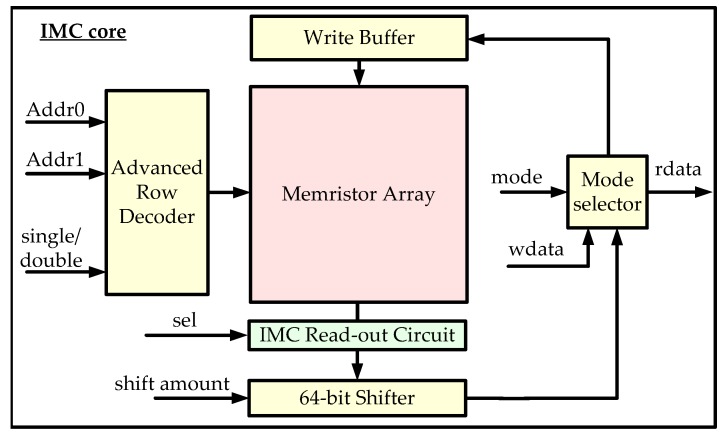
IMC core architecture.

The IMC core is designed to implement the IMC instructions. It consists of an advanced row decoder, a write buffer, a memristor array, an IMC read-out circuit, a 64-bit shifter, and a mode selector, as shown in Figure 3.

The read-out circuit is specially designed to implement the IMC logic instructions. The memristor array stores the data which participate in the IMC computations. These two modules are indispensable for IMC and are described in Section 3.2.2. The rest of the IMC core includes assistant circuits, which help to implement the IMC instructions and the control of the IMC core.

The advanced row decoder can either activate two row addresses simultaneously to execute IMC logic instructions or only one address to execute read/write instructions. The 64-bit shifter implements the 64-bit circular shift operation, and is disabled when other operations are performed. The mode selector decides whether the data are loaded out to the registers or written to the memory (either 64-bit data or 320-bit data) for CP and CPA. The write buffer is used when the data are written to the memristor array. A selection signal is sent to the Bitline (BL) calculator inside the IMC read-out circuit to determinate the IMC logic type. It should be noted that some control circuits are not shown in Figure 3 for conciseness.

#### 3.2.2. IMC Memristor Array and Read-Out Circuit

**Figure 4:**
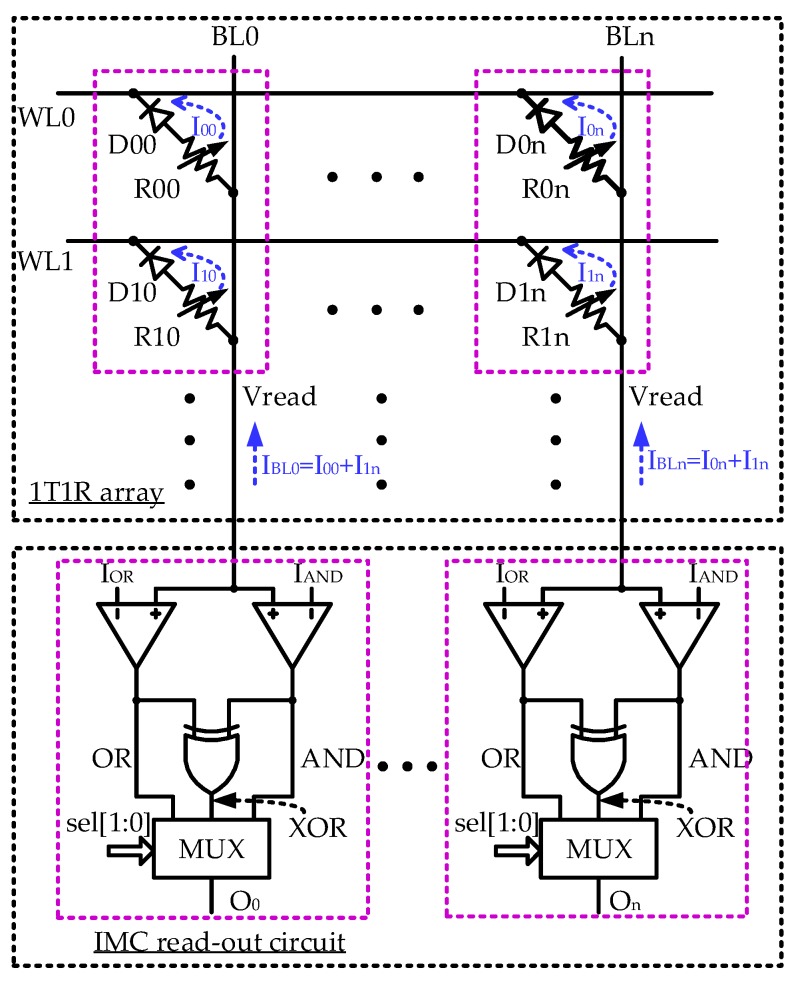
Memristor array with IMC read-out circuit.

In-memory computing implements all the 320-bit bitwise logic operations including AND, OR, and XOR operations in the hash algorithm using memristor-based IMC technology. As shown in Figure 4, a one-diode-one-memristor (1D1R) crossbar array is proposed with the IMC read-out circuit to realize the logic operations. The diode helps to restrain the disturbance of sneaking current to write/read, and logic operations; the memristor features unipolar set and reset operations. Using the diode as the selector, the 1D1R cell achieves higher density than the 1T1R cell [19]. Moreover, the diode selector and the memristor can both be integrated in the back end of line (BEOL) of the standard CMOS process. Therefore, the IMC core can be physically stacked by placing peripheral circuits on the substrate and lower interconnect metals, and the 1D1R crossbar array on the middle or upper interconnect metals. This can save area further, in accordance with the low-cost requirement of IoT devices.

The data are written into the 1D1R array by the processor in advance. The operation table of the 1D1R memristor array is shown in Table 4. Here, Vset/Vreset/Vread stand for the set/reset/read voltage of the memristor, and Vt is the threshold voltage of diode selector. The low-resistance state (LRS) of the memristor stands for logic “1”, while the high-resistance state (HRS) stands for logic “0”. To perform IMC, two selected wordlines (WLs), e.g., WL0 and WL1, are activated while applying proper read voltage (Vread) on the bitlines, e.g., BL0–BLn. The sum of currents along the same bitline (BL), e.g., *I*_BL0_ and *I*_BLn_, are compared with two reference currents, *I*_OR_ and *I*_AND_. The HRS is usually 10 times larger than the LRS [10], meaning that *I*_LRS_ >> *I*_HRS_, where *I*_HRS_ and *I*_LRS_ stand for the typical read currents for HRS and LRS, respectively. Therefore, the typical values of *I*_OR_ and *I*_AND_ can be set as 0.5 × *I*_LRS_ and 1.5 × *I*_LRS_. When *I*_SUM_ is larger than *I*_OR_, the signal OR becomes logic “1”, implying that at least one of the two activated memristors along the same bitline is the LRS. When *I*_SUM_ is larger than *I*_AND_, the signal AND becomes logic “1”, implying that both activated memristors along the same bitline are the LRS. By sending the results of OR and AND to an XOR gate, the XOR result is obtained at the output O_0_–O_n_. According to the control signal sel[1:0] from the assistant logic circuit, the corresponding result is written back to the 1D1R array in the next clock cycle. To perform 320-bit operations, this work adopts a 20-kb memristor array with 64 rows and 320 columns.

## 4. IMC Compiling Policy and Data Allocation Method

### 4.1. IMC Compiling Policy

In a traditional general processor, it is up to the software programmers to decide how to store the data needed, and the compiler to decide where to store them [20]. However, when it comes to IMC instructions, the programmer also has to decide whether to perform the computation with Arithmetic Logic Unit (ALU) or with IMC, requiring a special compiling policy. In addition, as mentioned in Section 3, only data in the same column and different rows can perform IMC logic operations; thus, IMC requires a different data allocation policy.

When a 32-bit vector is to be calculated with another 32-bit vector, ALU can finish this process in one clock cycle if the data are already cached in the registers, but IMC needs two clock cycles. This indicates that IMC consumes more processing time than ALU when performing simple logics. However, if both vectors are originally in the memory, ALU needs two additional clock cycles to load them out, and another clock cycle to store them into the memory if needed. This consumes more time than IMC. More generally, for a certain part of the algorithm with A 32-bit inputs, N steps of basic 32-bit operations, and Y 32-bit outputs (including long-lifetime intermediate results that cannot be cached in general registers), ALU takes (A+N+Y) clock cycles to process, whereas IMC needs 2N. Therefore, ALU should be used to perform calculations when

*A* + *N*+ *Y* < 2*N*, (7)

i.e.,

*N* > *A* + *Y*. (8)

Similarly, if the vectors are 64-bit long, ALU needs at least 2–6 clock cycles to finish this operation, but IMC needs only two clock cycles anyway; thus, IMC should be used to perform the calculations. This works better for vectors with widths larger than 64 bits. To sum up, for 32-bit vectors, ALU performs better when Equation (8) is satisfied, and, for 64-bit or longer vectors, IMC is always better.

### 4.2. Data Allocation Method for SHA-3

In terms of data allocation, IMC logics require any data processed to be in the same columns and different rows, and then data in the same row are handled simultaneously. Therefore, it is required that data placed in the same columns should frequently be operands of IMC operations, and data in the same row should share the same IMC operations frequently.

**Figure 5:**
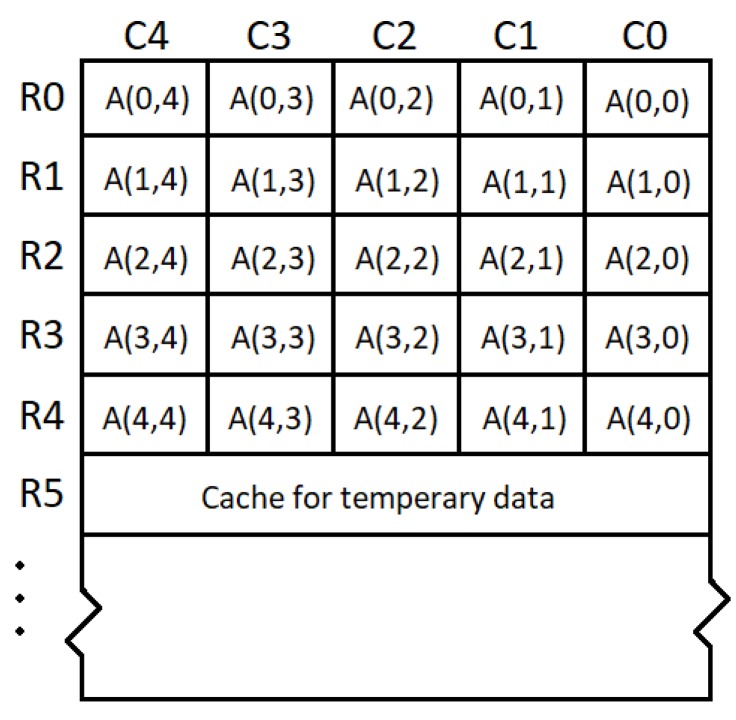
Data mapping of the 1600-bit state array.

Considering the regular features in Keccak-*f* permutation and the general compiling policy, we decided to adopt the data allocation method as shown in Figure 5. The 1600-bit state array is placed in row addresses R0–R4, and five 64-bit words are located in each row address with column address C0–C4, denoted as A(x,y). The five permutation steps are processed as below.

a. θ step

Perform XOR operations and get the XOR result of R0–R4, and then put the result in R5. Copy the result in C0–C4 of R5 to all columns in R6–R10 by performing CPA operations. Perform SHIFT operations on R6–R10 with the result placed in R11–R15. Then, XOR operations with the result placed in R0–R4 can be performed to finish the θ step.

b. ρ and π step

The ρ step and π step can be processed in a mixed way. Copy all data from R0–R4 to R5–R9; then, perform SHIFT operation to get the rotated value (stored temporarily in R10) and CP operations to update the data in R0–R4.

c. χ step

Copy data in C0–C4 of R0 to R5–R9 by CPA operations, and perform NOT, AND, and XOR operations in succession and update R0. Repeat this process five times so that all R0–R4 rows are updated. Note that the NOT operation can be performed by XOR with an all-1 vector.

d. ι step

In the ι step, there are lots of frequently used data and few long vectors; thus, ALU is used to perform this operation, and the instructions can be given by a C compiler.

## 5. Evaluation

### 5.1. Evaluation Methods

The proposed RISC-V processor with IMC for the Keccak algorithm was evaluated against the baseline one without IMC in terms of area, processing time, and energy consumption. The evaluation was carried out using the 28-nm process parameters.

For area evaluation, the control and operation module in Verilog hardware description language (HDL) format was firstly compiled by a Synopsys design compiler to acquire the equivalent gate count, which was then multiplied by the size of two-input NAND gate, i.e., NAND2, in the 28-nm process to get the corresponding area. The total area of the processor was calculated by summing the area of the control and operation module, the area of two working SRAM memories, and the area of the 20-kb IMC module.

**Figure 6:**
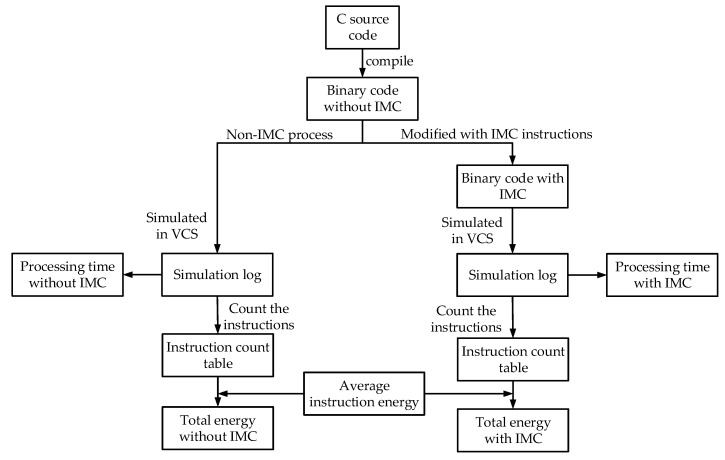
Evaluation method for processing time and energy consumption.

Figure 6 gives the evaluation method for processing time and energy consumption. To evaluate the processing time, the Keccak process was simulated in a Synopsis VCS Verilog simulator [21]. A 7-byte binary string was adopted as the test input. By simply compiling the C source code of the Keccak algorithm, the baseline processor could give the SHA-3 value through a non-IMC method. Then, by adding IMC instructions into the compiled machine codes of Keccak algorithm, the IMC-extended processor could give the SHA-3 value through an IMC method. The processing time can be acquired from the simulation log files. The energy evaluation was based on the simulation results of processing time. Firstly, the executed instructions in both cases were counted from the simulation log files separately. Then, based on the average energy consumption of individual instructions, the total energy consumption could be obtained by weighted summation.

### 5.2. Area Overhead

**Figure 7:**
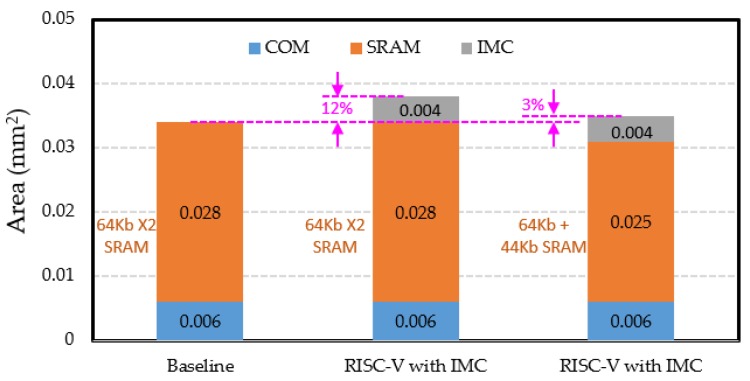
Area comparison between the baseline and the proposed RISC-V with IMC.

The equivalent gate count of the control and operation module, i.e., COM, was compiled to be about 110 K. Given the size of NAND2 to be 0.9 μm × 0.56 μm, the area of COM was about 0.006 mm^2^. The two working SRAM memories both had a capacity of 64 kb. The SRAM cell size was 0.12 μm^2^ and the total area of two working SRAMs was 0.028 mm^2^ [22]. For the IMC module, the count of IMC read-out circuits was required to be as many as 320 to support 320-bit bitwise logic operations. Assuming that each IMC read-out circuit had a size of 2 μm × 4 μm, the total area of IMC read-out circuits was 0.0026 mm^2^. The area of the advanced row decoder was estimated to be 0.001 mm^2^, i.e., 50 μm × 20 μm. The areas of the other circuits in the IMC module were relatively small and were estimated to be 0.0005 mm^2^. By 3D stacking, the 20-kb memristor array of the 1D1R cell would not bring additional area cost. To sum up, the area of the IMC module was about 0.004 mm^2^. Figure 7 shows the area comparison of the baseline and the RISC-V processor with IMC. The IMC module brings an area overhead of about 12%. However, the memristor array in the IMC module also plays the part of data cache; thus, the capacity of SRAM memory for data can be reduced, alleviating the area overhead. When the capacity of SRAM memory for data is reduced by 20 kb, the total area is reduced by about 0.003 mm^2^, and the area overhead is decreased to only 3%.

### 5.3. Performance Improvement

**Figure 8:**
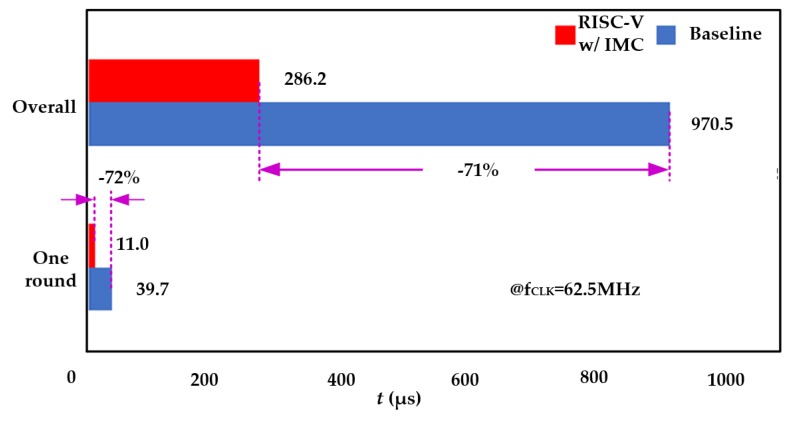
Comparison of processing time for the Keccak algorithm.

The processing time of the baseline RISC-V processor and the proposed one with IMC can be easily given by the simulator. The simulation was performed at a clock frequency of 62.5 MHz. Since our IMC technology accelerates each round in the Keccak-*f* permutation, both the processing time in one round and the overall process were considered, as shown in Figure 8. The processor can achieve over 70% improvement in terms of processing time for both one round and the overall process.

### 5.4. Energy Reduction

The average energy consumption for different operations was firstly characterized in the 28-nm process, as shown in Table 5. The energy consumed by SRAM read or write was similar. The 1D1R memristor cell consumed more energy than SRAM for read and write due to large active currents. Furthermore, the write operation of the memristor was even more energy-consuming than the read. Since the IMC logic was performed mainly by the read operation, the IMC readout circuits and other peripheral circuits still brought additional energy consumption by about 50%. All the parameters were closely relevant to the circuit design techniques and can be further optimized.

The average energy for each instruction is described in Table 6. The energy of ALU instruction refers to the energy consumed by the control and operation module to fetch an instruction from instruction SRAM, and then to decode and execute the instruction. The energy of SRAM read/write refers to the energy consumed by the normal ALU instruction and the energy to read/write 32-bit data from/to the data SRAM. The energy of IMC read/write refers to the energy consumed by the normal ALU instruction and the energy to read/write 32-bit data from/to the memristor array. The energy of IMC CP refers to the energy consumed by the normal ALU instruction and the energy to read 64-bit data from the memristor array and then write it to another address in the memristor array. The energy of IMC CPA refers to the energy consumed by the normal ALU instruction and the energy to read 64-bit data from the memristor array and then write it to five addresses in the same row of the memristor array. The energy of IMC logic refers to the energy consumed by the normal ALU instruction and the energy to perform 320-bit IMC logic and then write the 320-bit result back to the memristor array. The energy of 320-bit IMC SHIFT refers to the energy consumed by the normal ALU instruction and the energy to read 320-bit data from the memristor array and then write it back to the memristor array after shifting. It should be noted that IMC instructions usually consume more energy than ALU and SRAM read/write (R/W) instructions. With the development of memristor technology, the power consumption can be expected to decrease.

**Figure 9:**
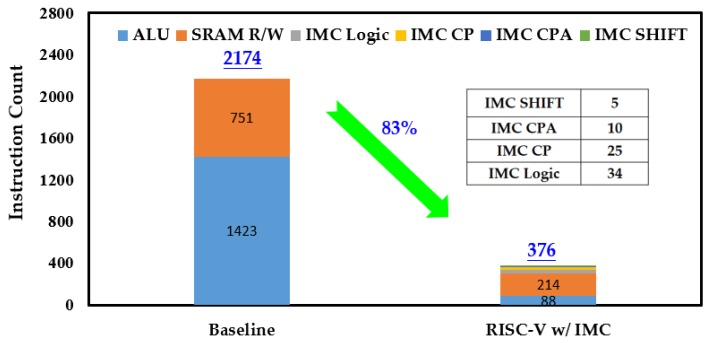
Comparison of instruction count in one round of Keccak-*f* permutation.

**Figure 10:**
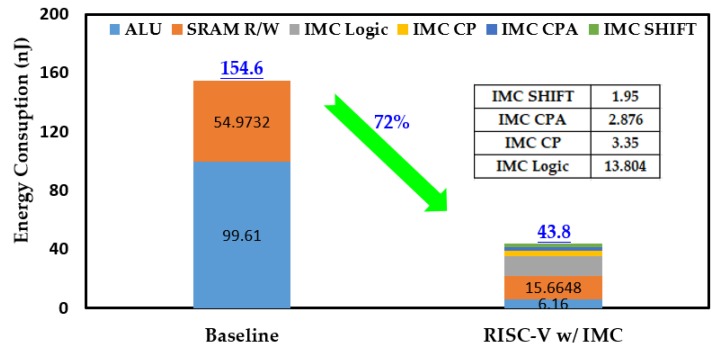
Comparison of energy consumption in one round of Keccak-*f* permutation.

**Figure 11:**
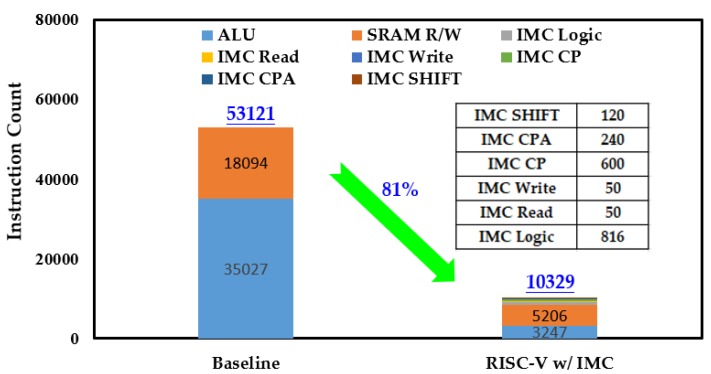
Comparison of instruction count in overall Keccak process.

**Figure 12:**
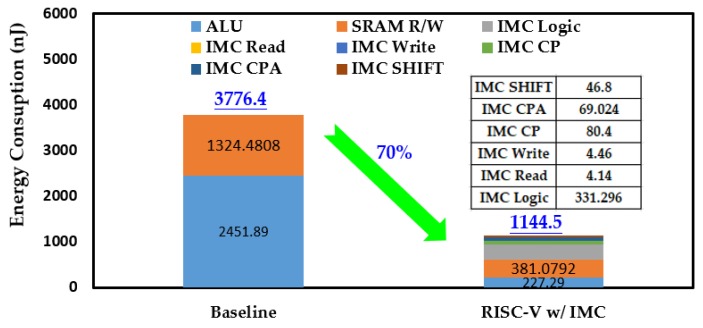
Comparison of energy consumption in overall Keccak process.

Like the processing time, both the energy consumption in one round of Keccak-*f* permutation and the overall process were considered. Figure 9 gives the comparison of instruction count of the baseline RISC-V processor and the one with IMC. In one round of Keccak-*f* permutation, the instruction counts of ALU and SRAM R/W were greatly reduced and the total instruction count was reduced by 83% after introducing IMC. The reduced instructions mean less data transferred between the memory and the ALU and also less workload for the ALU. As a result, the energy consumption in one round of Keccak-*f* permutation was reduced by 72%, as shown in Figure 10. Among the IMC instructions, the IMC logic brought the most energy consumption, accounting for more than 60%. Although the IMC instructions are generally energy-consuming, remarkable energy reduction was still achieved owing to the sharp reduction in instruction count. The reductions in instruction count and energy consumption for the overall Keccak process show similar trends to one round of Keccak-*f* permutation, achieving reductions of 81% and 70% after introducing IMC, respectively, as shown in Figure 11 and Figure 12. It should be noted that our simulation adopted a 7-byte binary string as the Keccak input, and, if the input data were infinitely long, the energy improvement tended to approximate to the extent of one round of Keccak-*f* permutation.

### 5.5. Comparison with Mainstream Mining Platforms and SRAM-Based IMC

Table 7 gives a comparison of the proposed memristor-based IMC with mainstream mining platforms and SRAM-based IMC. For the CPU, GPU, and ASIC, we selected i5 2500k from Intel, Tesla S1070 from Nvidia, and Antminer S4 from Bitmain, all of which were implemented in the 28/32-nm technology node. The performance was measured by the hash rate, i.e., hash operations performed in one second (H/s). The SRAM-based IMC was evaluated using the same method with the memristor-based one in this work.

From the comparison, two key points are worth mentioning. Firstly, although the CPU/GPU/ASIC provided higher performance than the SRAM/memristor-based IMC RISC-V, the latter consumed much less power (more than four orders of magnitude), which satisfies the low-power requirement of IoT applications. The large power of the CPU/GPU/ASIC also brings the need for cooling facilities. Moreover, a great number of IoT devices can also be coordinated to acquire high performance [4]. Secondly, memristor-based IMC brings less area cost (>50%) than SRAM-based IMC. Currently, SRAM-based IMC is more energy-efficient (~30%) than memristor-based IMC. The reason is that the write and read operations of the memristor consume much larger current. However, with the development of memristor technology, it is believed that the power consumption of the memristor will decrease. Moreover, the nonvolatility of the memristor enables the IMC module to fully power-off without data loss during standby mode, helping to reduce the total standby power.

## 6. Conclusions

Security for private information on IoT devices is becoming increasingly important. The hash function used in blockchain helps to ensure information security, as well as data integrity. However, the corresponding hardware in IoT devices is challenging when realizing the complex hash algorithm owing to a low energy budget. This paper proposes combining in-memory computing with the highly extensible RISC-V for lower-power hash algorithm implementation. Remarkable improvements in both performance and energy consumption were achieved with limited area overhead. Further work may involve general compiling techniques to help the processor with IMC to realize diverse functions.

## Figures and Tables

**Table 1:** Five steps in Keccak-*f* permutation.

Step	Equations	Main Process
θ	(1)	Massive 64-bit and 320-bit bitwise XOR operations, a few 64-bit shift operations
ρ	(2), (3)	Massive 64-bit shift operations and data copying
π	(4)	Massive 64-bit data copying
χ	(5)	Massive bitwise 320-bit logic operations (XOR, OR and AND)
ι	(6)	Massive operations on one 64-bit binary string

**Table 2:** In-memory computing (IMC) applications in Keccak-*f* permutation. XOR—exclusive or; SHIFT—64-bit shift operation; CPA—copy to all columns; CP—64-bit data copying operation.

Step	IMC Involved
θ	XOR, SHIFT, CPA
ρ	SHIFT, CP
π	CP
χ	XOR, OR, AND, CPA
ι	None

**Table 3:** IMC-adapted instruction definition list.

Bit	31–30		29–25	24–20	19–15	15–13	12	11–7	6–0
XOR	00		A1	A2	BA	I/R		A0	Custom0
OR	01		A1	A2	BA	I/R		A0	Custom0
AND	10		A1	A2	BA	I/R		A0	Custom0
SHIFT	11		A1	SA[5:0]	BA	I/R	SA[6:0]	A0	Custom0
Normal read	Imm[11:0]				rs	Reserved		rd	Custom1
Normal write	Imm[11:5]			rs2	rs1	Reserved		Imm [4:0]	Custom2
CP and CPA	0	Flag	A1	A2	BA	I/R	Col[5:0]		Custom3

**Table 4:** Operation table of one-diode-one-memristor (1D1R) memristor array for IMC. HRS—high-resistance state; LRS—low-resistance state.

Operation Mode	Wordline (WL)		Bitline (BL)	
	Selected	Un-Sel	Selected	Un-Sel
Set (HRS→LRS)	0	Vset	Vset + Vt	0
Reset (LRS→HRS)	0	Vreset	Vreset + Vt	0
Logic (Read)	0	Vread	Vread + Vt	0

**Table 5:** The average energy consumption for different operations in the 28-nm process.

Operation	Energy (pJ)
ALU	70
SRAM read/write	0.1/bit
memristor read	0.3/bit
memristor write	0.6/bit
memristor logic	0.45/bit

**Table 6:** The average energy consumption for each instruction in the 28-nm process.

Instruction	Main Actions	Energy (pJ)
ALU	Fetch, decode and execute the instruction	70
SRAM read/write	ALU, 32-bit SRAM read/write	73.2
IMC read	ALU, 32-bit memristor read	82.8
IMC write	ALU, 32-bit memristor write	89.2
IMC CP	ALU, 64-bit memristor read and write	134
IMC CPA	ALU, 64-bit memristor read and 320-bit memristor write	287.6
IMC Logic (AND, OR, and XOR)	ALU, 320-bit memristor logic and write	406
IMC SHIFT	ALU, 320-bit memristor read and write	390

**Table 7:** Comparison of memristor-based IMC with central processing unit (CPU), graphics processing unit (GPU), application-specific integrated circuit (ASIC), and SRAM-based IMC.

Mining Platform	Performance (H/s)	Active Power (Watts)	Energy Efficiency (J/H)	Area (mm^2^)
CPU (i5 2500K) [23]	4.80 × 10^4^	90	1.88 × 10^−3^	large scale
GPU (Tesla S1070) [23]	1.55 × 10^8^	8.00 × 10^2^	5.16 × 10^−6^	large scale
ASIC (Antminer S4) [23]	2.00 × 10^9^	1.40 × 10^3^	7.00 × 10^−7^	large scale
SRAM-based IMC	1.03 × 10^3^	8.80 × 10^−4^	8.50 × 10^−7^	3.50 × 10^−2^
Memristor-based IMC	1.03 × 10^3^	1.17 × 10^−3^	1.14 × 10^−6^	5.50 × 10^−2^
